# Improving transcriptome *de novo* assembly by using a reference genome of a related species: Translational genomics from oil palm to coconut

**DOI:** 10.1371/journal.pone.0173300

**Published:** 2017-03-23

**Authors:** Alix Armero, Luc Baudouin, Stéphanie Bocs, Dominique This

**Affiliations:** 1 Montpellier SupAgro, UMR AGAP, Montpellier, France; 2 CIRAD, UMR AGAP, Montpellier, France; 3 South Green Bioinformatics Platform, Montpellier, France; INRA, FRANCE

## Abstract

The palms are a family of tropical origin and one of the main constituents of the ecosystems of these regions around the world. The two main species of palm represent different challenges: coconut (*Cocos nucifera* L.) is a source of multiple goods and services in tropical communities, while oil palm (*Elaeis guineensis* Jacq) is the main protagonist of the oil market. In this study, we present a workflow that exploits the comparative genomics between a target species (coconut) and a reference species (oil palm) to improve the transcriptomic data, providing a proteome useful to answer functional or evolutionary questions. This workflow reduces redundancy and fragmentation, two inherent problems of transcriptomic data, while preserving the functional representation of the target species. Our approach was validated in *Arabidopsis thaliana* using *Arabidopsis lyrata* and *Capsella rubella* as references species. This analysis showed the high sensitivity and specificity of our strategy, relatively independent of the reference proteome. The workflow increased the length of proteins products in *A*. *thaliana* by 13%, allowing, often, to recover 100% of the protein sequence length. In addition redundancy was reduced by a factor greater than 3. In coconut, the approach generated 29,366 proteins, 1,246 of these proteins deriving from new contigs obtained with the BRANCH software. The coconut proteome presented a functional profile similar to that observed in rice and an important number of metabolic pathways related to secondary metabolism. The new sequences found with BRANCH software were enriched in functions related to biotic stress. Our strategy can be used as a complementary step to *de novo* transcriptome assembly to get a representative proteome of a target species. The results of the current analysis are available on the website PalmComparomics (http://palm-comparomics.southgreen.fr/).

## Introduction

Palms are a plant family represented by more than 2,500 species in 184 genera distributed in tropical and subtropical regions of America, Africa and Asia [1]. As indicated by Couvreur et al. [1], evolution of palms is closely linked to rain tropical forest history. Several species of this family are keystone species, i.e their size and abundance play a major role in shaping their ecosystem.

The most popular species of palms is coconut (*Cocos nucifera L*). This species is the main source of income of multiple tropical communities and it played a role unequaled in the colonization of the pacific islands [2]. Coconut production in the coming decades faces several challenges to plant breeders as a result of climate change and market demand for increasing yield and diversification of products. Genetic and genomic knowledge in this species is still minimal; to date NCBI database reports only 1,008 ESTs sequences and 3,903 proteins [3] and several transcriptomes [4–6]. On the contrary, oil palm *(Elaeis guineensis L*.*)* receives greater attention because of its high productivity and profitability, which made palm oil the most common oil on the world market [7]. The interest in oil palm led to the sequencing of the genome of a palm species for the first time [7]. Genomic resources have also been developed for other palms such as the date palm [8] offering the opportunity to resolve fundamental questions of palms biology and opening new routes to the genetic improvement of these species [8–9]. For example, was the whole genome duplication experienced by *Elaeis guineensis* [10] shared by coconut and did it affect gene families diversification?. Improving the coconut genomic resources quality would help the understanding of the evolutionary history of this species compared to other palms and consider possible routes for a future coconut plant breeding.

In this context, translational genomics, i.e translating genomic knowledge from a model plant to a closely related orphan or non-model plant [11] is a mean to deepen the understanding of coconut biology by using advances on the oil palm research. Here, we propose a bioinformatics methodology that improves coconut transcriptome data by using an oil palm proteome. The methodology responds to several of the limitations of the transcriptomic data, by exploiting the sequence similarity between species, and offers as product a coconut proteome that can be used for different types of analysis such as phylogenetics, evolution of gene families, diversity, among others.

Transcriptomic data obtained by the new generation sequencing technologies (NGS) are a powerful and rapid mean to obtain valuable information on the genetic composition and expression of a given genome [12]. However, given the complex task of assembling the coding sequences without a reference genome, *de novo* transcriptomes undergo several drawbacks [13]. Wang et al, identified two types of errors that can occur in the clustering of expressed sequence tags (EST). Type I error occurs when ESTs of the same gene are not grouped into the same cluster, while type II error appears when ESTs of different genes are grouped in the same cluster. The type I error involves the fragmentation of genetic sequences, whereas type II represents chimeric sequences [13]. Transcriptomic data are also difficult to assemble because of a redundancy problem: it is not obvious to separate alternative transcripts of a gene, alleles, and gene copies of a multigenic family. These factors prevent obtaining a correct proteome that can be used in downstream analysis. A recent study showed that Trinity, a software dedicated to *de novo* assembly, has a high resilience to chimerism, while this software and some others with high performance (CLCscaf-S, SOAPtrans-S) have a significant rate of fragmentation as a result of the difficulty of assembling coding sequences with either low or high levels of expression [14].

Some studies have explored translational genomics to improve *de novo* transcriptome in non-model organisms. In order to identify the allelic variation present in the transcriptome of 14 different genotypes of *Lolium perenne*, a methodology based on orthology was developed [15]. This strategy called Orthology Guided Assembly (OGA) identifies the transcripts that can be orthologous to genes of a group of reference species and reduces redundancy by using CAP3 software. Another approach with CAP3 software is the scaffolding translation mapping (STM) method that maps reads and transcripts to the proteome of a reference species [16]. CAP3 is considered to be a conservative tool, demanding a high similarity between sequences [17]. This feature can prevent this tool to capture all the redundancy in *de novo* contigs or perform the scaffolding when the degree of overlap between the fragments is not long enough to be identified by this program. Thus CAP3 may not be completely adequate to reduce contig redundancy and their scaffolding.

**Fig 1:**
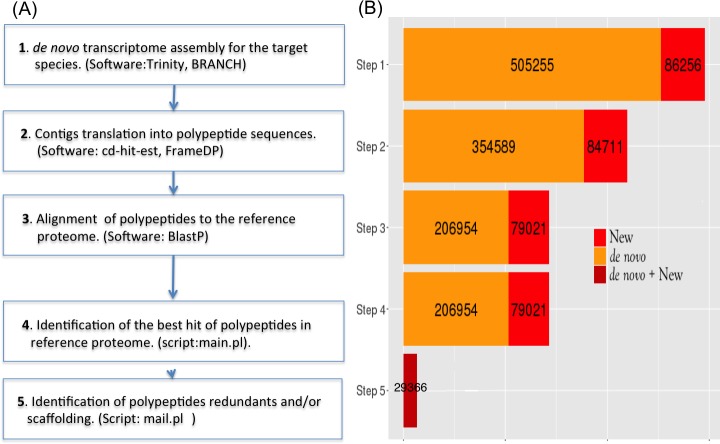
Methodology and results obtained on coconut. (A) Principal steps of the methodology to recover a target proteome, using transcriptomic data and a reference proteome from a related better studied organism. (B). Number of sequences generated on coconut by our methodology starting from four coconut transcriptomes. Sequences in red represent the number of new sequences found by BRANCH. Sequences in orange represent *de novo* transcriptomes assembled by Trinity. Protein products (PP) generated at step 5 (in dark red) combine information from both categories of sequences.

In order to overcome these hindrances, we developed a methodology that reduces the fragmentation and redundancy of *de novo* transcriptomes by providing a workflow to build a reference proteome in a neglected species (coconut). The main characteristics of this workflow are: i) Identify new contig sequences with BRANCH software [18], aligning reads and *de novo* transcriptome to a closely related well-studied organism (oil palm). ii) Translate a large pool of polypeptide sequences from transcriptome with FrameDP software [19]. iii) Process the alignments of these polypeptides to an oil palm proteome (Fig 1).

We validated our methodology by reversing the logic of model species and reassembling a well-known proteome from RNA-Seq data. *Arabidopsis thaliana* is a model species and has been the first plant genome to be sequenced [20]. Genomic resources are also available on related species such as *Arabidopsis lyrata* and *Capsella rubella* [21, 22]. These species have diverged around 12 and 23 M.Y.A respectively from *Arabidopsis thaliana* [23]. In order to evaluate the performance of our methodology and the influence of the reference species on the results, we assembled the *Arabidopsis thaliana* proteome from transcriptomic data, using *Capsella rubella* and *Arabidopsis lyrata* proteomes as references. This analysis, applied to a well-known genome, indicated that the workflow can produce a proteome with a high precision and respond significantly to the problems of redundancy and fragmentation.

This validation allowed us to apply our methodology to coconut and to produce an improved proteome, made available as a comparative genome hub in reference to oil palm pseudochromosomes. We finally assessed the functional representation of the coconut proteome, product of the workflow.

## Material & methods

### Workflow used to improve a target transcriptome by using a reference genome

Our methodology, starting from short nucleotide sequence reads generated by New Generation Sequencing technologies (NGS) of a transcriptome, involves five steps summarized in Fig 1A.

#### Step 1. *de novo* assembly of a target transcriptome

NGS transcriptomic studies are generally represented by paired-end reads. The target paired-end reads were trimmed using Trimmomatic V033 [24]. Transcriptomes were assembled with the software Trinity [25].

We used the BRANCH software as a first strategy to improve *de novo* transcriptome by translational genomics. BRANCH can identify novel contigs, extend incomplete contigs and join fragmented ones by using partial or related genomic sequences [18]. In the current analysis we used BRANCH with default parameters to find new contigs by using the reads and target contigs assembled at step 1 and the mRNA of the reference species as a related genomic sequence.

#### Step 2. Contigs translation into polypeptide sequences

The target transcriptomes and the new sequences found by BRANCH software were pooled. Identical sequences (full-length and fragments) were removed with cd-hit-est V4.6.4 software [26], retaining the longest sequence as the representative of the redundant group. These transcripts were translated into polypeptide sequences with FrameDP V1.2.2 software [19] and default parameters, using UniProtKB viridiplantae proteins [27] and relevant reference databases [28–30].

#### Step 3. Alignment of polypeptides to reference proteins

The target polypeptides of the previous step were aligned to the proteins of the reference species using BLASTP [31]. The alignments were processed to recover high-scoring segment pair (HSP) which do not overlap over more than 1 amino acids (aa). The alignments were subjected to the following algorithm to identify potential chimeric sequences:

1. All HSPs with a at least of 50% identity between the target polypeptides and the proteins of the reference species were identified.
2. If at least two disjoint regions of the target polypeptide aligned to two different proteins of the reference species, and none of these alignments covered more than 70% of the target polypeptide, this sequence was tagged as chimeric.

#### Step 4. Selection of the target polypeptides best hit in the reference proteome

The selection of the best hit was based on two parameters of quality of alignment: the identity and the coverage of target polypeptides. It was more stringent for small sequences to ensure accurate results in the last step. The best hit choice involved three steps:

1. An alignment was retained if the identity was at least 80% and the coverage of the target polypeptide was at least 70% (with a coverage of the reference protein below 40%) or 60% (with a coverage of the reference protein equal or above 40%).
2. The list of alignments was ordered according to the coverage of target polypeptide, the identity of alignment and coverage of the reference protein in that order of importance.
3. The best alignment of a target polypeptide was considered as the one at the top of the ordered list.

#### Step 5. Identification of redundant polypeptides and scaffolding

This step aimed to reduce redundancy and join target polypeptides into a single protein product (PP) by using the homology to the reference proteome. This step was done by processing the best hits to the reference proteome through the following steps:

1. All target polypeptides having the same reference protein as best hit were identified.
2. The non-redundant target polypeptides were identified, i.e. the proteins which had the best coverage of a region of the reference protein. All others target polypeptides that had a lower coverage of this region of the reference protein were labeled as redundant.
3. The non-redundant target polypeptides were sorted according to their first position of alignment to the reference protein.
4. If there was only one non-redundant polypeptide it was declared as the protein product. If there was more than one non-redundant polypeptides, the positions of alignment of two consecutive target polypeptides were compared. If the positions were overlapping, the rightmost aa in the alignments shared by the two target polypeptides was identified and this served as a position of cut and join for the two sequences. Otherwise, the target polypeptides were joined with *n* times”X” where *n* represents the distance between the alignments of these polypeptides.

Steps 4 and 5 and the identification of potential chimeric sequences were executed by a series of scripts that can be obtained at http://palm-comparomics-pj.southgreen.fr/Download.html/Scaff.RRed.tar.gz. A more detailed explanation of the use of this script is found in S1 File.

### Precision assessment using *A*. *thaliana*

Precision was evaluated by two parameters: sensitivity and specificity, assessed by comparing the proteins obtained with the workflow in *A*. *thaliana* to an “expected group” of proteins of this species.

The “expected group” of *A*. *thaliana* proteins were the sequences in TAIR database [32], which met two conditions, i) there is evidence that these proteins are present in the polypeptides derived from contigs and ii) these proteins have significant homology to the *A*. *lyrata* or *C*. *rubella* proteomes. We counted as evidence of the presence of a protein of *A*. *thaliana* database in the polypeptide sequences produced at step 2, the fact that polypeptides were significantly aligned (*i*.*e*. identity > 60, target coverage > 70) to *A*. *thaliana* proteins with BLASTP software. To meet the second condition, the proteins of *C*. *rubella*/*A*. *lyrata* were aligned to proteins of *A*. *thaliana* that met the first condition, these alignments were filtered (identity > 60, target coverage > 70), and *A*. *thaliana* proteins retained were considered the “expected group”.

After applying the workflow, we aligned the resulting *A*. *thaliana* protein product (PP) with BLASTP software to the “expected group” of proteins defining three categories:

**True Positive (TP)**: A *A*. *thaliana* PP has a significant alignment to a protein of the “expected group”.

**False Positive (FP)**: A *A*. *thaliana* PP does not have a significant alignment to a protein of the “expected group”.

**False Negative (FN)**: A protein of the expected group does not have a significant alignment to a *A*. *thaliana* PP.

An alignment was considered significant if the identity was at least 60% and the coverage of the protein from the “expected group” was minimum 70%. The categories enabled to calculate the measures of sensitivity and specificity.

The sensitivity is the ability of the methodology to recover the proteins of the expected group and was calculated by:

Sn=TP/(TP+FN)

The specificity decreases when the methodology suggests proteins that are not present in the expected group. This measure was calculated by:

Sp=TP/(TP+FP)

The behavior of these measures was studied by varying the parameters of identity and coverage in the alignments of step 4 of the workflow. There are three alignment parameters: the first represents the identity, the second is the coverage for target polypeptides covering more than 40% of the protein of expected group (reference) and the last is the coverage of target polypeptides covering 40% or less of the protein of the expected group. These parameters varied from 0/0/0 to 80/75/85.

### Impact of scaffolding step in length, coverage and identity of the *A*. *thaliana* proteins

The increase in length of *A*. *thaliana* PPs was assessed by analyzing the coverage and the identity of alignments of polypeptides before and after step 5 to *A*. *thaliana* proteins TAIR 10 when *A*. *lyrata* was used as reference species.

### Functional annotation in coconut and comparative analysis of metabolic pathways

Gene Ontologies: The gene ontology terms of coconut polypeptides before and after step 5 were determined using PRIAM software [33]. The distribution of proteins in the categories of level II in the three graphs (cellular component, molecular function, biological process) was compared between coconut proteins and *Oryza sativa* proteins [27] with WEGO software [34].

The enrichment analysis of coconut PPs which consist exclusively of polypeptides derived from new contigs (contigs founded by BRANCH software not identical at the sequence level to the contigs assembled by Trinity) was carried out with GOstats packages version 2.38.0 [35] in R [36]. A concise visualization of the results was performed with the software Revigo [37].

Biosynthetic pathways: The nomenclature of KEGG encyclopedia of coconut polypeptides before and after step 5 was inferred by InterproScan software [38]. The metabolic pathways of coconut polypeptides was identified with Pathway Tools [39] and compared to *O*. *sativa* pathways [40].

### Transcriptome representation on a comparative genome hub

A website called PalmComparomics http://palm-comparomics.southgreen.fr/ was developed as a representation of the current analysis. This site is based on in-house Apache tools and tools of the Generic Model Organism Database (GMOD) project [41]. For instance, coconut scaffolded polypeptide matches along oil palm genome are viewable using JBrowse [42]. We exploited the facilities of Bio::DB::SeqFeature::Store schema to create a MySQL database with the annotations of coconut polypeptides from gff3 files. The website also included the CocoCyc Pathway/Genome Database (PGDB) containing the predicted metabolic pathways.

### Data

#### Workflow application in coconut

The methodology was applied to coconut transcriptomic data recovered from NCBI [4–5]. The first dataset corresponds to a transcriptomic analysis of endosperm, embryo and leaf of a dwarf variety (SRX518095, SRX534380 and SRX534428) [4], while the second represents transcriptome data from a mix of tissues from Hainan tall cultivar (SRX198908) [5]. The new contigs were identified by using mRNAs of oil palm recovered from the NCBI Reference Sequence Database (RefSeq), annotation release 100 [28]. The translation into polypeptide sequences of coconut contigs was performed using as reference database the proteins of *Elaeis guineensis* [28], uniprotKB viridiplantae [27], *Phoenix dactylifera* [29] and *Musa acuminata* [30]. Finally, *Elaeis guineensis* proteome (RefSeq 100) [28] was used in step 5 of the workflow to produce a coconut proteome.

#### Sensitivity and specificity test

We applied the workflow to three transcriptomic datasets of *A*. *thaliana* recovered from NCBI: SRR1640366, SRR1640370, SRR2895761 [43, 14]. The proteins of “expected group” of *A*. *thaliana* was derived from TAIR10 database [32]. The *Arabidopsis lyrata* [21] and *Capsella rubella* [22] proteomes were recovered from phytozome v. 11 [44].

#### Functional annotation

The gene ontology terms of *Oryza sativa* v 7.0 were recovered from UniprotKB [27]. The biosynthetic pathways of *Oryza sativa* were recovered from PlantCyc [40].

A complete description of the software and the parameters used in each of the steps of the workflow is described in the document S1 File. This document also presents how you can obtain the main results of our analysis of the website PalmComparomics (http://palm-comparomics.southgreen.fr/).

Data used in the present paper can be retrieved at the following sites:

Transcriptomic data at NCBI site (https://www.ncbi.nlm.nih.gov/)

Cocos nucifera: datasets SRR1273180, SRR1273070 SRR606452, SRR606452

Arabidopsis Thaliana: datasets SRR1640366, SRR1640370, SRR2895761

mRNA and proteins: see links provided in refs 28, 29 and 30

Other data available at the following sites

Uniprot gene ontology rice: ftp://ftp.ebi.ac.uk/pub/databases/GO/goa/proteomes/23240.O_sativa_indica.goa

Uniprot viridiplantae: http://www.uniprot.org/taxonomy/33090

Phytozome v11.0: https://phytozome.jgi.doe.gov/pz/portal.html

PlantCyc sites: ftp://ftp.plantcyc.org/Pathways/Data_dumps/PMN10_June2015/oryzacyc_pathways.20150608

Data produced during the present work can be downloaded at http://palm-comparomics.southgreen.fr/Download.html or at https://github.com/armerovillanueva/PalmComparomics

## Results

### Our methodology offers a high precision when generating a new proteome

Before applying our methodology to coconut, we tested its precision on a well studied species: *Arabidopsis thaliana*. In this study several proteomes were obtained by varying the filtering parameters in step 4 using *Arabidopsis lyrata* and *Capsella rubella* as reference species. These proteomes were aligned to an expected group of proteins in *A*. *thaliana* and sensitivity and specificity were calculated.

The expected group for *A*. *thaliana*, i.e. proteins with significant alignments to polypeptides derived from contigs and presenting homology to *A*. *lyrata/C*.*rubella* respectively, consisted of 22,015 proteins with *A*. *lyrata* and 20,200 proteins with *C*. *rubella*.

The methodology described in Fig 1A was applied to three groups of transcriptomic data of *A*. *thaliana* (S1 Table). A pool of 121,076 contigs were obtained from *de novo* assembly, BRANCH and elimination of redundancy of identical sequences (full-length and fragments) (step 1, Fig 1A). We found a translation to polypeptide sequence for 101,688 contigs with FrameDP software (step 2, Fig 1A). These polypeptides were aligned and filtered to different thresholds of identity and coverage to *A*. *lyrata/C*. *rubella* proteomes (step 3 and 4, Fig 1A). Finally, elimination of redundancy and scaffolding for each of these groups of polypeptides was performed according to the last step of workflow (step 5, Fig 1A).

We tested several filtering parameter sets (see below and S1 Table) and develop here the results for the 80/60/70 parameter set. At the end of step 4, we recovered 77,541 and 70,803 *A*. *thaliana* polypeptides when *A*. *lyrata* and *C*. *rubella* were used respectively as references. The numbers of final protein products (PP) after step 5 were 19,943 (*A*. *ly*rata) and 18,615 (*C*. *rubella*) resulting in a reduction of redundancy by factors of 3.88 and 3.80 respectively.

**Fig 2:**
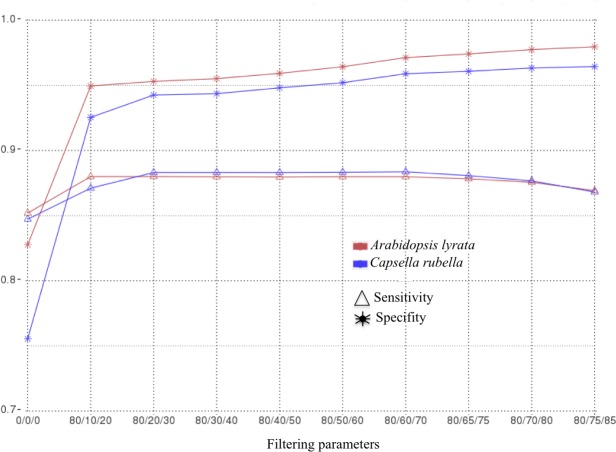
Analysis of sensitivity and specificity in *Arabidopsis thaliana*. Curves of sensitivity (triangles) and specificity (stars) in *Arabidopsis thaliana* for different filtering parameters (step 4) using *Arabidopsis lyrata* (in red) and *Capsella rubella* (in blue) as reference species. The filtering parameters consist of three digits. The first represents the identity, the second is the coverage for target polypeptides covering more than 40% of the protein of expected group (reference), and the last is the coverage of target polypeptides covering 40% or less of the protein of the expected group.

When varying the filtering parameters, the sensitivity and specificity varied in a similar way between the two species used as reference (Fig 2). In both analyses, it was evident that an initial filtration of the alignments performed at step 4 is required to improve the specificity. This trend was especially notable in *C*. *rubella* where a first filtration increased the specificity from 0.75 to 0.93. After this initial point, there was a slight increase of specificity and a stable behavior of sensitivity as filtration parameters became more stringents until the parameters 80/60/70 (Fig 2). In this combination, sensitivity reached 88% and specificity was higher than 96%. When using more stringent filtering parameters, sensitivity decreased slightly, as a consequence of an increase of false negatives (S1 Table, Fig 2).

We compared the number of true positives found with the two reference species in filtering parameters (80/60/70). 16,569 polypeptides were shared by the two analyses, 2,801 were specific to *A*. *lyrata* analysis and 1,281 to *C*. *rubella*.

### The methodology improves the polypeptide sequences derived from *de novo* transcriptome

We assessed the performance of the methodology to recover the full-length sequence of a protein ensuring the amino acid identity. The polypeptide sequences of step 4 and 5 of the methodology were compared in terms of length, coverage and identity to the corresponding proteins of *A*. *thaliana* in TAIR10.

**Fig 3:**
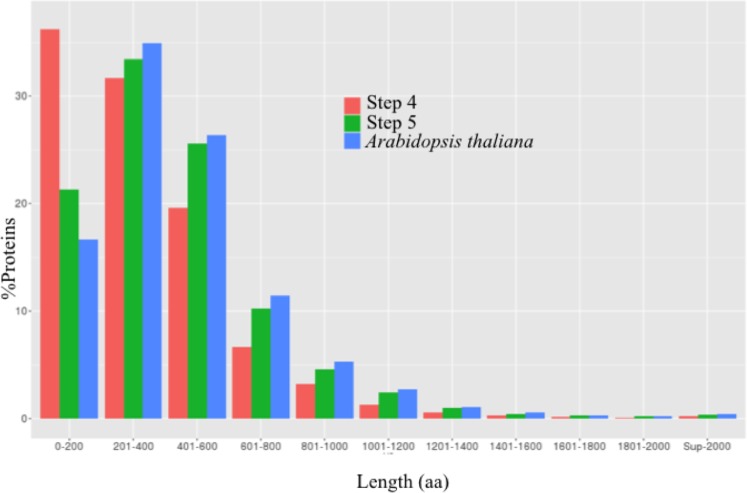
Increase on the length of *A*. *thaliana* PPs. Length distribution of polypeptides in step 4 (in orange) and 5 (in green) and distribution of *Arabidopsis thaliana* TAIR10 proteome (in blue). Protein lengths are indicated as discrete length ranges, from 0–200 to >2000 aa.

The comparison between the ranges of protein lengths between step 4 and 5 of workflow showed that step 5 reduced the percentage of short polypeptides while increasing the percentage of the large polypeptides, approaching protein length distribution of *A*. *thaliana* TAIR10 (Fig 3). Before step 5, 36.2% of the polypeptide sequences had a length lower than 200 aa, while after step 5 this percentage dropped to 21.3%. In all other ranges of length, the percentage of sequences was increased after step 5, concentrating 78% of sequences between 200 to 2,000 aa. In the sequences with lengths greater than or equal to 2000 aa, the percentage was doubled after step 5 (0.18–0.36%), approaching to that observed in *A*. *thaliana* (0.43%). Finally, the average length of the polypeptide sequences was increased from 339 to 429 aa.

The identity of alignments to proteins from *A*. *thaliana* TAIR10 before and after step 5 remained unchanged (99%), while the average coverage of protein in database was increased from 0.87 to 0.90. To describe the impact of the methodology on the coverage, the distribution of this trait was divided into ranges, and amount of polypeptides that changed their coverage range after the scaffolding of step 5 was calculated (S2 Table). 53.0% (10,745) polypeptides before scaffolding had already a 100% coverage to proteins in TAIR10. Among the 9,106 remaining polypeptides, 26.4% (2,425) moved to a higher range of coverage after scaffolding. The polypeptides that were improved had an average increase of 18% in coverage, i.e. an average gain of 145 aa in length. The percentage of polypeptides with 100% coverage was increased by 7.4% (1,481). Only 1.2% of proteins moved to a lower range.

### The methodology largely eliminates chimeric sequences

We evaluated the number of chimeric sequences at the main steps of the methodology. We identified 1,272 *A*. *thaliana* polypeptides sequences that are rated as chimeric sequences when aligned to the proteome reference of *A*. *lyrata* (step 3). In order to an exact evaluation of the amount of potential chimeric sequences the polypeptides were also aligned to the proteome reference of *A*. *thaliana*. As a result, the amount of chimeric sequences was reduced to 745, i.e. 0.61% of all polypeptides. Moreover, only 77 of these 1,272 sequences were retained in step 4 after filtration (80/60/70). In the step 5, 54 of these potential chimeric sequences were declared redundant, 5 were cut and joined to other sequences in the scaffolding process and the other 18 sequences represent complete PPs.

On the other hand, the scaffolding process could create new chimeric sequences by binding polypeptides belonging to different proteins. For this reason we evaluated the amount of PPs that could be considered as potential chimeras. When PPs were aligned to *A*. *lyrata* proteome, 47 potential chimeric sequences were identified. When PPs were aligned to the *A*. *thaliana* proteome, the amount of chimeric sequences was 142, representing 0.71% of all PPs.

### The methodology provides an improved *Cocos nucifera* proteome

We applied the methodology in order to obtain a proteome of coconut using oil palm as model species.

In order to obtain a better representation of the coconut proteome, we worked with two different transcriptomes [4, 5]. The contigs obtained with Trinity and BRANCH from the four different groups (20,847 new/264,349 *de novo* dwarf embryo, 29,122/185,242 dwarf endosperm, 27,625/88,707 dwarf leaf and 23,283/89,133 tall tissue mix) were pooled and identical sequences (full-length and fragments) were eliminated, yielding 591,511 sequences (Fig 1B, step 1). Of these contigs, 14% (86,356) were identified by BRANCH software. 74% (439,300) of contigs were translated into polypeptides (step 2) sequences and 48% (285,975) of these had significant alignments to oil palm proteins (step 3 and 4). At step 5, these polypeptides sequences aligning to oil palm proteins were integrated into 29,366 coconut protein products (PPs). Each of these polypeptides represented on average 9,74 sequences of step 4 and 24% (7,058) of them were improved by scaffolding.

35% polypeptides (153,325) were lost between step 2 and step 3 because they had no significant hits in oil palm proteome used in this analysis. Among them, 7,716 had significant alignments (identity > = 80%, coverage > = 70%) to proteins in TrEMBL database. The remaining 145,609 polypeptide sequences had an average length of 106 aa in comparison to a length of 336 aa in polypeptides that had significant alignments to oil palm or other species. These polypeptides without significant alignments may represent orphan genes or artifacts of *de novo* transcriptome due to the presence of genomic DNA in the sample and/or non-coding RNA.

We quantified the degree of chimerism in coconut sequences. Among all the polypeptides that could be derived from the transcripts (step 3), 6,332 were identified as potential chimeric sequences (1.44%). Only 153 of these potential chimeric polypeptides were retained in step 4. 130 of these potential chimeric sequences were declared redundant, 7 intervened in the scaffolding process and 16 represented full PPs sequences. Finally, the alignment of coconut PPs to oil palm proteome revealed the existence of 23 PPs that could be considered chimeric.

### The methodology identifies more coconut contigs

In this section we consider a new contig as a sequence that has been found by the software BRANCH and is not identical at the level of nucleotide composition to contigs obtained with Trinity. These new sequences represent contigs with evidence in RNAseq data and that were assembled using the oil palm mRNA in step 1 of the methodology.

In step 4 there were 79,021 polypeptide sequences derived from new contigs. 14,258 of these sequences were used in step 5 to obtain coconut PPs, while the remaining polypeptides obtained from the new contigs were identified as redundant. 11,196 of these 14,258 polypeptides were involved in the scaffolding of 4,059 coconut PPs, and 3,062 represented each a complete coconut PP sequence. This represented an average gain of 122 aa in the corresponding polypeptides.

Our workflow produced 1,246 coconut PPs that were completely new sequences, i.e these sequences would not have been recovered from the polypeptides derived from *de novo* assembly contigs with Trinity. Usually, these polypeptides represented only part of the reference proteins: when they were aligned to the corresponding proteins of reference species, the average coverage was significantly lower (0.60) than the coverage of all the proteome product of the workflow (0.89).

### The methodology allows the identification of full-length protein products

**Fig 4:**
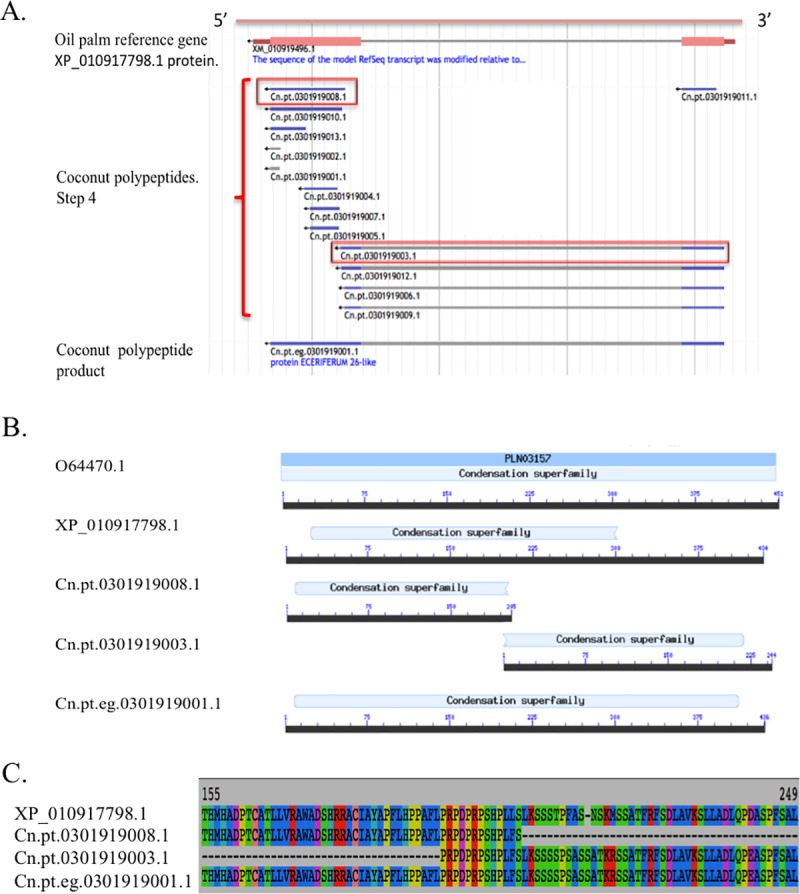
Scaffolding of coconut polypeptides homologous to oil palm ECERIFERUM 26-like protein. (A) Snapshot of oil palm protein, coconut polypeptides and coconut PP in ‘Palmcomparomics’ Jbrowse. Polypeptides within red squares were used for scaffolding of coconut PP. (B) Functional domain identified in coconut sequences and oil palm protein. The sequence O64470.1 of *Arabidopsis thaliana* is representative of the condensation superfamily. (C) Alignment between oil palm protein, coconut polypeptides and coconut PP around the overlap region.

An example of scaffolding of coconut polypeptides is depicted in Fig 4. The oil palm protein used as a reference is coded as XP_010917798.1 [28]. This protein is homologous to sequences known as ECERIFERUM 26-like, enzymes involved in epicuticular wax biosynthesis and pollen fertility [45]. In Fig 4A, the gene coding XP_010917798.1 is presented in its genomic context, with the representation of alignments of coconut polypeptides in step 4 and after step 5.

In this example, several redundant coconut polypeptides sequences overlapped to two sequences (Cn.pt.0301919003.1 and Cn.pt.0301919008.1, Fig 4A) which were scaffolded and, together, represented the entire homologous coconut PP. The protein domains were identified with CDD software [46] and are represented in Fig 4B. Polypeptides of oil palm and coconut shared a ‘condensation’ domain in their sequences. This domain catalyses a condensation reaction to form peptide bonds in nonribosomal peptide biosynthesis [46]. The scaffolding step accurately joined the two coconut polypeptides (Fig 4C), thereby obtaining the complete condensation domain after scaffolding, despite the fact that the quality of the oil palm sequences does not allow to recover the whole domain in oil palm.

### The comparison of coconut proteome to public data shows the ability of our methodology to reduce transcript fragmentation

We compared the proteome obtained in the current analyses with the polypeptides derived of two published transcriptomes [4, 5]. 57,175 transcripts recovered from Fan et al [5] and 153,852 from Huang et al [4] were translated into 37,743 and 76,553 polypeptides (66% and 49% of the transcripts respectively). The percentage of translated transcripts in our methodology was 66%.

These polypeptides datasets were aligned to rice proteome. The polypeptides of step IV have the highest amount of best-hits in rice (19,470), followed by Coconut PPs derived from our methodology (13,237), compared to 11,018 in Huang et al [5] and 9,535 in Fan et al [4]. The average identity for all datasets was similar, at around 66%. The S3 Fig shows the distributions of the coverage of rice proteins for the different datasets. While the distribution is approximately flat for polypeptides obtained from Huang et al, the other three distributions were characterized by a large peak at high values of coverage of rice proteins (>90%). The distribution of PPs (step 5) presents the highest concentration of sequences with good coverage, followed by the polypeptides of step 4, which in turn have a slightly better distribution than that observed in Fan et al.

### The methodology allows an accurate functional representation of coconut transcriptome

We annotated the coconut PPs by identifying the gene ontology (GO) terms, including the 1,246 sequences that were never identified before (see above). The resulting GO terms at level II were compared to the functional annotation of *Oryza sativa* (the model species of monocots).

At least one GO term was assigned to 186,185 (42%) of coconut polypeptides in step 4, to 19,628 (67%) coconut PPs and to 22,674 (53%) *O*. *sativa* proteins. S1 Fig reveals the similarity between the distribution of polypeptides in the GO categories before and after the last step of workflow, indicating that the elimination of redundancy does not alter the functional representation of this species. Similarly, the distribution of coconut PP in gene ontology categories was similar to the distribution of *O*. *s*ativa. The categories of biological processes with a high percentage of genes in rice and coconut were related to the basic metabolism necessary to cell survival (S1 Fig).

“Cellular processes” is one of the major categories in the coconut PPs and *O*. *sativa* proteome (GO:0009987). This category includes the processes involved in reproduction, growth and cell death. Another category with an important representation in the proteomes of these species is “metabolic process” (GO:0008152). This category represents the metabolic processes of biosynthesis and degradation of primary and secondary metabolites. Biological processes related to cell cycle regulation (GO:0043473) or the regulation of transcription activity and phosphorylases (GO:0065007) also have an important representation in rice and coconut sequences. Among the molecular functions with an important representation, we found “binding activity” (GO:0005488) and “catalytic activity” (GO:0003824), the latter covering a broad spectrum of enzymes such as transferases, transposase, ligases, lyases etc.

Nevertheless, the distribution of proteins in the gene ontology categories revealed interesting differences between coconut and rice, this comparison suggests a significant degree of specialization of rice proteins. For example, no coconut PP was found for the category “viral process” (GO:0016032), i.e. a multi-organism process in which a virus is a participant (S1 Fig). There are eight rice proteins belonging to this category, including CYP701A8, an enzyme involved in phytoalexins biosynthesis [47]. We aligned these proteins to the coconut PPs. The best hit in coconut for rice protein CYP701A8 was Cn.pt.eg.0300260001.1 (Id = 58%, Cv = 96%). Cn.pt.eg.0300260001.1 was aligned to the non-redundant proteins NCBI database with smartblast [31]. The best alignment was obtained for ALF95896.1, a coconut mRNA derived polypeptide (Id = 99%, Cv = 99%). Putting aside palms, the best alignment for coconut PP was NP_197962.1 (Id = 63%, Cv = 87%), a ent-kaurene oxidase from *A*. *thaliana* involved in gibberellin biosynthesis [48].

### New sequences have functions related to biotic stress

It was possible to identify at least one Gene Ontology term in 748 of the 1,246 proteins derived exclusively from new contigs (S3 Table). This group of sequences was enriched in four main biological processes: response to biotic stimulus, protein phosphorylation, organelle organization and glucosamine-containing compound metabolism. In several of these groups the genes formed clusters on the chromosomes.

Response to biotic stimulus is especially important in coconut, which is subject a variety of lethal diseases [6, 49]. In the group of polypeptides involved in responses to biotic stimulus (GO:0009607) and defense response (GO:000695) 14 coconut PPs were identified as homologous to oil palm proteins, annotated as pathogenesis-related 1-like protein and MLO-related protein (*Mildew Locus O*) [50]. Five of the nine proteins of oil palm annotated as pathogenesis-related 1-like proteins formed a cluster on chromosome 14. Five coconut polypeptides were derived from oil palm protein annotated as MLO-related protein and had significant alignments to various *A*. *thaliana* proteins belonging to the MLO gene family that acting as susceptibility factors towards fungi causing the powdery mildew (S3 Table) [50].

“Protein amino acid phosphorylation” (GO:0006468) is a diverse group of 72 serine/threonine kinases. We identified 10 coconut PPs derived from oil palm references bearing the annotation “G-type lectin-like receptor S-serine/ threonine-protein kinase”. In oil palm, 7 of these proteins are part of two clusters, one on chromosome 1 and the other one on chromosome 2. Another important group of phosphorylases found in the new sequences are cysteine-rich protein receptor-like kinases. Four of these proteins also form a cluster on chromosome 2 of oil palm. Other kind of phosphorylases cover a wide spectrum of functions, e.g. Leucine-rich repeat receptor kinases (LRR-RKS) [51] and strubbelig receptor family (S3 Table) [52].

An important group of 25 coconut PPs had similarity to oil palm proteins with functions related to organelle organization (GO:0006996). Some are involved in the assembly of mitochondria (mitochondrial fission proteins) and peroxisomes biogenesis. Other proteins do a negative regulation of these morphogenetic processes by preventing polymerization of actin filaments or by promoting depolymerization of this molecule (F-actin-capping protein). Among these proteins, some also have a negative control over the organization of the cytoskeleton and mitosis (mitotic spindle checkpoint protein MAD2-like protein). Finally, some of these new proteins are histones or NAP1-related protein 2-like with functions related to chromatin modification [27].

A small group of three coconut proteins with glycoside hydrolase functions were related to chitinase of oil palm (GO:0006040). The Pfam annotation indicated that two of these proteins belong to the family hydrolase Glycoside 19, involved in plant defense against fungi and other pathogens. The other protein belongs to the family hydrolase Glycoside 18 (S3 Table) [53].

### The coconut proteome has a large number of specialized metabolism pathways

The presence of the major classes of biosynthetic pathways of coconut polypeptides was assessed, and this result was compared with the pathways identified in *O*. *sativa* (S2 Fig). The coconut proteome presents 567 metabolic pathways, of which 453 (80%) belong to the “biosynthesis” class, while rice has 415 of this type representing 85% of total metabolic pathways in this species. S2 Fig presents the quantity of pathways subclasses in each species that are shared or exclusive to one species, in the main classes of biosynthetic pathways. In most subclasses, pathways shared by the two species are the most numerous. This was the case for carbohydrate biosynthesis, fatty acid and lipid biosynthesis, nucleosides and nucleotides biosynthesis (S2 Fig). A different trend was represented by secondary metabolites biosynthetic class, where shared and specific pathways were represented by a similar number. At the other end of the spectrum, biosynthetic pathways of amines and polyamines were all species specific.

### PalmComparomics

In order to facilitate the access to genomic information in coconut, we developed the web site “PalmComparomics” (http://palm-comparomics.southgreen.fr/) that contains the main results of the current analysis. This web site, hosted by the Southgreen bioinformatic platform, offers several tools for data querying and visual analyses. PalmComparomics has a browser where oil palm genes and the mapping of coconut polypeptides to oil palm pseudo chromosomes are represented. Data can be retrieved for specific terms of gene ontology, Pfam domains, metabolism pathways, Interpro domains or sequence homology. PalmComparomics provides a BLAST tool that allows the user to align sequences against the coconut polypeptides and contigs. The polypeptides and proteins products of our methodology can be downloaded in FASTA format. It is also possible to retrieve a GFF3 file with gene ontology annotations, and Interpro or Pfam domains of coconut polypeptides.

## Discussion

### Fragmentation, redundancy and chimerism

The methodology developed in this work corrected transcripts fragmentation to a large extent. In the methodology validation made in *A*. *thaliana*, a substantial percentage of polypeptides with shorter sequences than expected were improved, and this gain in length resulted in recovering the entire sequence of the polypeptides in many cases. In coconut, a quarter of all PPs of the workflow were obtained by scaffolding. The relevance of the scaffolding process was reflected in the comparison of our coconut proteome to polypeptides obtained from Fan et al, [5] and Huang et al, [4] transcriptomes. In fact, the last step of the methodology increased the frequency of polypeptides that have high coverage of the rice proteins. The scaffolding could recover the full functional domain of a protein, although it was initially divided into polypeptides fragments, and regardless of the completion of the protein used as reference. This plasticity of the methodology to resolve the correct union of the fragments in most cases was the result of our scaffolding strategy on polypeptide sequences.

Our strategy differs from previous approaches using CAP3 like OGA or STM by using the protein of reference species as a guide for the scaffolding based on the alignment positions of target polypeptides. It is more flexible than these approaches because using reference proteins avoids the need to determine the similarity and coverage between the polypeptides to be joined.

These strengths of the methodology respond to the potential drawbacks of the fragmentation in downstream analysis, such as the reliable assessment of homology [54] and errors in multiple alignments [55].

Our methodology applied to coconut generated a comprehensive group of protein products that are representative of polypeptides derived from a large number of contigs aligning to the proteome of a reference species. Reduction of redundancy is an important factor in functional and evolutionary analysis. Ono et al.[56] showed that reducing redundancy in transcriptomes improves detection of differentially expressed genes. Redundancy could be a problem in identifying orthologs and paralogs, defining multiples alignments and constructing distance matrix between sequences [57]. This new coconut proteome could therefore lead the way to more in-depth genomic evolution analysis in the palm group.

The intensity of fragmentation and redundancy is related to the species under study. Reducing redundancy and scaffolding in *A*. *thaliana* analyses was clearly lower than in coconut. Although this could result from the quality of *A*. *thaliana* genome annotation or the large number of contigs assembled in coconut, it may also suggest some impact of biological factors explaining these results. Below we discuss some of these biological factors.

Our methodology is heavily shielded against chimeric sequences. Our analysis showed that, in the first steps, the percentage of chimeric sequences can mount beyond 1% of the total proteins that are obtained from the translation of the transcripts. However, using appropriate alignment quality parameters allows filtering out most chimeric sequences. Additionally, step V functions as another filtering point for the chimeric sequences. These could be declared redundant, or they could be cut in the process of scaffolding eliminating chimerism. As a result, only a small proportion of the final PPs are chimeric sequences, that users should analyze further attentively, since many of these apparent chimerisms may represent whole proteins that have additional sequence segments with minor alignments against other reference proteins.

### Filtration issues in comparative analysis

The analysis of sensitivity and specificity showed the dependence of these variables in relation to the filtering parameters (identity and coverage) used in step 4 of workflow. The selection of filtering parameters determines the similarity between the sequences of target species and species reference. Relaxed parameters may result in chimeric sequences at the scaffolding step, producing background noise (increasing the number of false positives) and decreasing slightly the specificity of the outcome. Conversely, very stringent parameters reduce slightly the sensitivity by unnecessarily eliminating correct protein product sequences (increasing the number of false negatives).

The precision analysis performed in *A*. *thaliana* suggests that a combination of parameters (80/60/70) represents a good compromise in both references species (*A*. *lyrata*, *C*. *rubella*). It acts as a turning point, where the gain in specificity resulting from more stringent parameters no longer compensates the loss of sensitivity. We used these parameters to present the final results in *A*. *thaliana* and in coconut.

### Choosing the right reference species

The choice of a reference species involves the assessment of several factors. Perhaps the most influential of these factors is the availability of good quality genomic resources in closely related species. Although the rate of publication of genomes increase at a high-speed [58], there are still major taxonomic groups in which there is a low amount of published genomes, likes palms. Another important aspect is the quality of the structural annotation of genomes In plants, some species like *Arabidopsis thaliana* have benefited from great attention in manual curation and experimental support of gene function [58].

Another factor that should be taken in consideration is the evolutionary divergence between the target species and the potential reference species. Two aspects come into play. The first one is the divergence time between the species under analyses. The second one is the rate of evolution in the considered species or taxon, which is determined by life history traits such as organism size, metabolism rate, the life cycle and the ecological niche [59]. In *A*. *thaliana*, the divergence time may be the main explanation to the observed differences in the number of expected proteins, protein products and true positives obtained with *A*. *lyrata* compared to *C*. *rubella*.

Conversely, coconut and oil palm diverged around 60 M.Y.A [60]. However, a very good genome conservation has been found among palm species as shown by the redundancy reduction and scaffolding. Genome sequencing of oil palm showed that 94.4% of the genes of this species are homologous to genes of *Phoenix dactylifera* supporting our observations [7]. This genome conservation may result from a longer generation time compared to *Arabidopsis* [61].

### Functional representation of coconut proteome

The comparison of the distribution of gene ontology terms among coconut polypeptides before and after step 5 of the workflow showed that the later reduces redundancy without loss of functional representation. This functional profile is characterized by a large number of proteins involved in cellular and metabolic processes with catalytic and binding activities.

Our functional profile for the coconut PPs is also similar to the distribution of transcriptomes published by Fan et al [5] and Huang et al [4]. As indicated by Huang et al, this profile is shared by other species of monocots like banana and pineapple [4]. It also applies to the recently published transcriptome of *Nypa fruticans* [9], the only monocot adapted to mangrove.

While there is a great similarity between the GO distribution of coconut and rice proteins, there are also differences that may indicate a lineage specific processes. We evidenced one of these differences with CYP701A8, a rice protein that acquired a new role in the biosynthesis of phytoalexins, proteins implicated in resistance to biotic stress [47]. Although it was possible to identify sequences with significant similarity to CYP701A8 in the coconut proteome, this coconut sequences presented a higher degree of similarity to dicots proteins with functions in the gibberellins biosynthetic pathway. These results would need confirmation because our proteome is not necessarily complete. Therefore, the absence of evidence of a protein sharing the same function as CYP701A8 is not evidence of its absence.

The similarity of proteomes of coconut and rice was also confirmed through the comparison of the respective biosynthetic pathways. These species have an important number of pathways in common. However, the data did not show pathways of amines and polyamines shared by these species. This result could reflect true biological differences, but it has to be viewed with caution because it may be the product of transcriptomic bias and/or the limitations of the automatic annotation by pathway tools.

Interestingly, coconut has a high proportion of pathways involved in specialized metabolism that are not shared with rice. Secondary metabolites are important in the interaction of the plant with its environment [62]. In a comparative analysis of the pathways through the major taxonomic groups of plants, Chae et al., [63] showed that angiosperms are enriched in biochemical reactions involved in specialized metabolism. Some of the gene families involved in specialized metabolism appeared to be under selection and derived from local duplications [63]. Our results could motivate further investigation on the structure and evolution of secondary metabolism pathways in coconut and other palms.

Finally, we found that the new sequences identified with BRANCH are enriched in functions involved in biotic stress and organelles morphogenesis. These sequences are fragments with low coverage of complete sequence of mRNA, suggesting that these fragments are part of mRNA degradation product. This should be investigated further because many sequences with roles in biotic stress were identified as being over-expressed in coconut phytoplasma infection [6]. This is the case for pathogenesis-related proteins, MLO family and chitinases. Functions involved in the morphogenesis of peroxisomes and mitochondria could also be related to this coconut infection, considering the role of these organelles in the metabolism of reactive-oxygen species and response to stress [64]. Our current work shows that oil palm genes that are homologous to coconut diseases resistances genes are grouped in genomic clusters. This observation suggests active evolutionary mechanisms for local duplication of these genes.

### Limits of the comparative analysis

There are other factors to be taken into account for the application of the methodology. The methodology cannot recover proteins that are absents in the reference species, but are present in the target species.

In *A*. *thaliana* analysis, *C*. *rubella* allowed to identify 1,281 PPs that were not initially revealed when *A*. *lyrata* was the reference species. This result suggests a complementary approach that would consist in using more than one reference species. For coconut, a complementary reference species could be *Phoenix dactylifer*a, which genome has been also sequenced [8]. This could prove useful if the main goal is to have an exhaustive protein set however, there is a risk to introduce false positives.

Other proteins that can be recovered from the polypeptides derived from contigs are the orphan genes or “taxonomically restricted genes” i.e. genes uniques to the species or with a narrow phylogenetic distribution. The average percentage of orphan genes in a species are between 5 and 15% in plants [65]. These proteins may be retrieved from the initial set of sequences as those which do not match to the reference proteome, but present a “canonic” coding sequence feature. They must be further characterized by BLAST for highly conserved short stretches on a large set of sequenced genomes [66] or by InterproScan. The sequences, initially identified by these bioinformatic methods as candidate orphan genes, could be verified by transcriptomic or proteomic studies. The ratio of non-synonymous vs. synonymous substitutions of the coding sequences could be an additional signal of a selection pressure acting on those genes [67].

Finally, one last point to consider in the results of the methodology is the allelic diversity. In our study we used different transcriptomes, originating from separate genetic resources. The combination of transcripts from several studies allows a better coverage of protein sequence product and complementarity that may exist between different studies at the tissue level. The protein product identified by our methodology could be used as a reference to explore sequence diversity at the next step. In order to take allelic diversity into account, one approach would be to align the sequences that have been identified as redundant to the reference protein and determine sequence polymorphism at the nucleotide level, with potential implications as genetic markers or impact on the three-dimensional structure of proteins and their functions.

## Conclusion

We developed a simple and effective method to improve protein sequences which can be obtained from a transcriptome by translational genomics. This methodology is an additional step to *de novo* assembly and solves the problem of fragmentation and redundancy of contigs. A more complete protein sequence enables reliable phylogenetic inference of the evolution of gene families. This analysis represents a first step before the expected publication of genomic sequence of coconut, offering a proteome that would help improving the structural genome annotation.

## Supporting information

S1 Table Specificity and sensitivity in *Arabidopsis thaliana*. The table shows the number of contigs (QC) or polypeptides (QP) obtained at each step of the workflow, as well as the sensitivity and specificity analysis in *Arabidopsis thaliana* using *Arabidopsis lyrata* and *Capsella rubella* as reference species. Results are indicated for several filtering parameters, indicated as three digits: the first represents the identity, the second the coverage for target polypeptides covering more than 40% of the protein of expected group, and the last is the coverage of polypeptides covering 40% or less of the protein of the expected group. The filtering parameters retained for coconut are highlighted in yellow.
(XLSX)

S2 Table Increased coverage of *Arabidopsis thaliana* polypeptides. The table represents the amount of polypeptides that change or maintain the coverage rank (BLASTP alignment) of the respective proteins of *Arabidopsis thaliana* of TAIR10, between step 4 and 5 of the methodology using *Arabidopsis lyrata* as a reference species.
(XLSX)

S3 Table Functional annotation of new protein products. The table provides original data for new coconut PPs (derived exclusively from contigs found by BRANCH software). Unique identifiers and coordinates on the oil palm pseudochromosomes allows to retrieve the sequences in our ‘palmcomparomics’ database. GO terms, Pfam, E.C numbers and annotation for the corresponding oil palm protein are indicated.
(XLSX)

S1 Fig Distribution of Gene Ontology categories in coconut and rice. The figure presents the distribution of rice proteins (in red), coconut polypeptides from step 4 (in grey), and protein products (PPs) from step 5 (in orange) in Gene Ontology terms for three main GO categories (cellular component, molecular function, biological process).
(TIF)

S2 Fig Biosynthetic pathways comparison between coconut and rice. The figure indicates the number of subclasses in the main classes of biosynthetic pathways that are shared (in blue) or specific to coconut (in orange) or rice (in green)
(TIF)

S3 Fig Coverage of the rice proteome by coconut proteins obtained with the current methodology compared to proteomes derived from public transcriptomes. The frequency distribution of four sets of coconut polypeptides are plotted according to their coverage of rice proteins.
(TIF)

S4 Fig Representation of the alignment of a new coconut PP alignment to oil palm protein reference and functional domains. (A). Visualization of the alignment of the polypeptides of step 4 and Cn.pt.eg.Un05781001.1 coconut PP in relation to the XP_010910661.1 oil palm protein in Jbrowse. (B). Functional domains of coconut polypeptides, PP and the protein reference of oil palm.
(TIF)

S1 File Supplementary Note. Detailed description of the parameters of the software used in the workflow and of the data produced by our research.
(DOCX)
