# Supplementary figures and images for: Improving transcriptome de novo assembly by using a reference genome of a related species: Translational genomics from oil palm to coconut
Source: PLoS One. 2017 Mar 23;12(3):e0173300. doi: 10.1371/journal.pone.0173300 (PMC5363918; doi:10.1371/journal.pone.0173300)

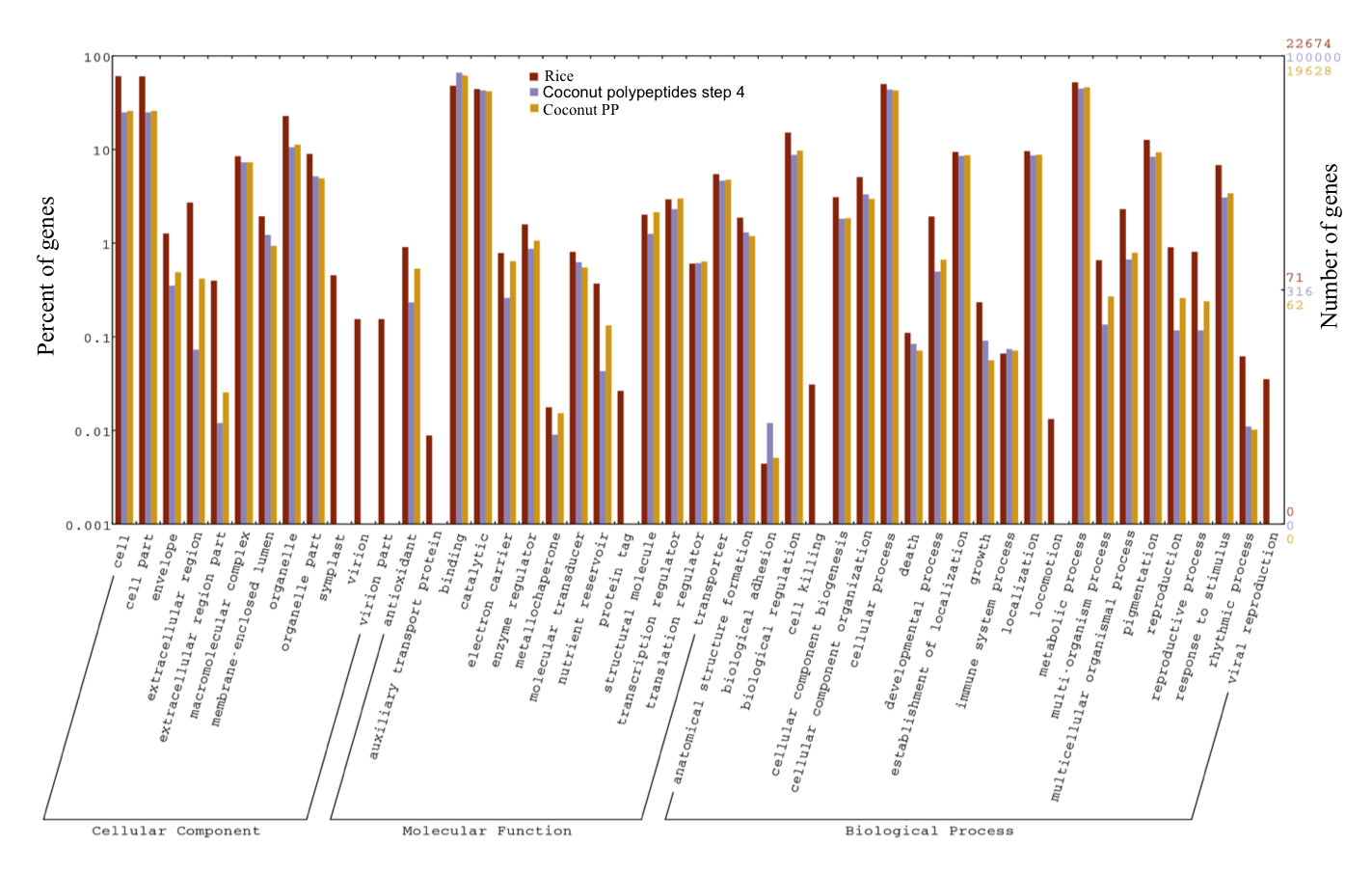

Supplement: S1 Fig — The figure presents the distribution of rice proteins (in red), coconut polypeptides from step 4 (in grey), and protein products (PPs) from step 5 (in orange) in Gene Ontology terms for three main GO categories (cellular component, molecular function, biological process). (TIF) [file pone.0173300.s004.tif]

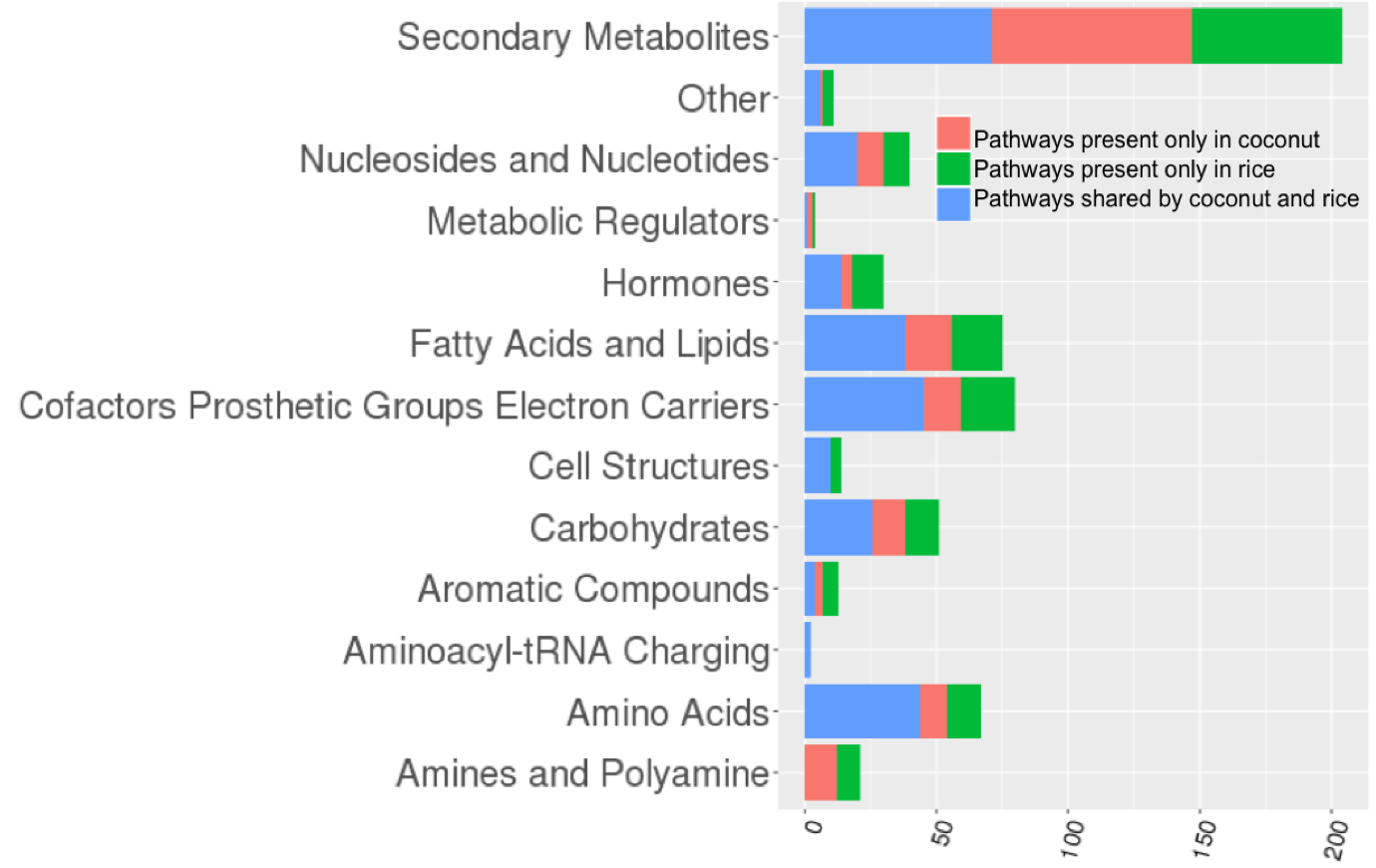

Supplement: S2 Fig — The figure indicates the number of subclasses in the main classes of biosynthetic pathways that are shared (in blue) or specific to coconut (in orange) or rice (in green) (TIF) [file pone.0173300.s005.tif]

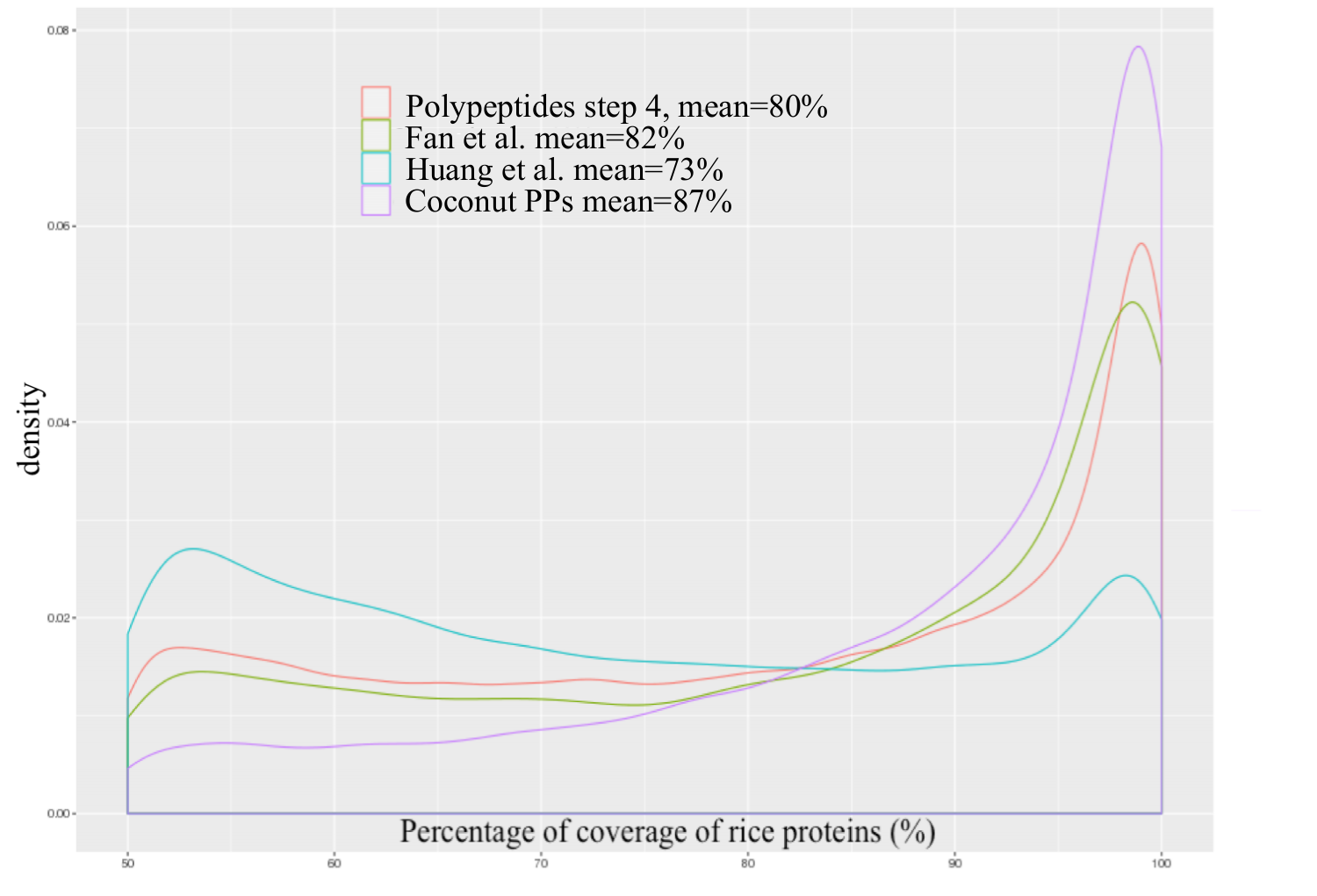

Supplement: S3 Fig — The frequency distribution of four sets of coconut polypeptides are plotted according to their coverage of rice proteins. (TIF) [file pone.0173300.s006.tif]

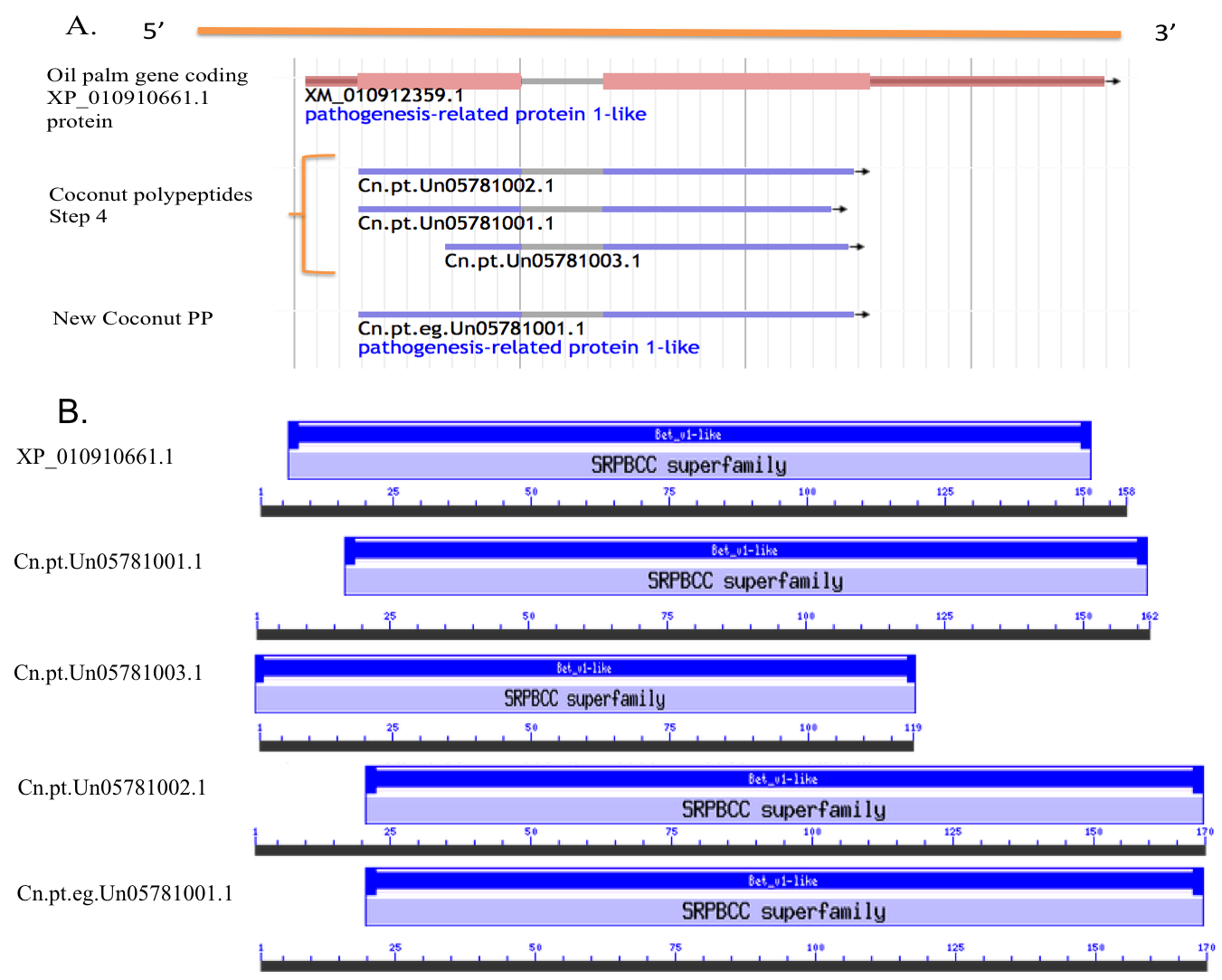

Supplement: S4 Fig — (A). Visualization of the alignment of the polypeptides of step 4 and Cn.pt.eg.Un05781001.1 coconut PP in relation to the XP_010910661.1 oil palm protein in Jbrowse. (B). Functional domains of coconut polypeptides, PP and the protein reference of oil palm. (TIF) [file pone.0173300.s007.tif]
