# Supplementary material for: Improving transcriptome de novo assembly by using a reference genome of a related species: Translational genomics from oil palm to coconut
Source: PLoS One. 2017 Mar 23;12(3):e0173300. doi: 10.1371/journal.pone.0173300 (PMC5363918; doi:10.1371/journal.pone.0173300)
Supplement: S1 File — Detailed description of the parameters of the software used in the workflow and of the data produced by our research. (DOCX) [file pone.0173300.s008.docx]

Improving transcriptome de novo assembly by using a reference genome of a related species: translational genomics from oil palm to coconut.

Alix Armero, Luc Baudouin, Stéphanie Bocs, Dominique This.

## Supplementary Note

In this supplementary note the main technical information about the workflow implemented during this work is presented, in order to improve transcriptome de novo assemblies of one species (e.g. coconut) according to reference omic data of a related species (e.g. oil palm). We provide the software and parameters used in each step of the workflow and we make an introduction to the scaffolding and reduction of redundancy scripts.

## Workflow software and parameters

### Step 1.

####

#### Read trimming.

1.1.1. Trimming.

Software: Trimmomatic V033 (1).

Cmd: java -jar trimmomatic-0.33.jar PE Reads1.fastaq Reads2.fastq

Output1.fastq Output.unpaired1.fastq Output2.fastq Output.unpaired2.fastq

ILLUMINACLIP:File.fa:2:30:10 LEADING:6 TRAILING:6

SLIDINGWINDOW:4:20 MINLEN:35

- Input

- Reads1.fastaq, Reads2.fastq. Paired-end reads in fastq format.

- Output

- Output1.fastq, Output2.fastq. Paired-end reads after trimming.

- Output.unpaired1.fastq, Output.unpaired2.fastq. One of reads in the pair was lost during the trimming.

- Parameters
- PE: Paired-end reads
- File.fa:2:30:10. Trimmomatic looks for the adapters specified in the File.fa file, allowing 2 mismatch, if a score of 30 is reached, the seeds are extended and clipped.
- LEADING:6. Cut bases off the start of a read with a quality below 6
- TRAILING:6. Cut bases off the end of a read with a quality below 6
- SLIDINGWINDOW:4:20. Scanning at the 5’ end and clips the read once the average quality within the window of 4 bases falls below of score of quality of 20.
- MINLEN:35. Only reads longer than 35 bp after trimming were kept.

1.1.2. Quality control of the trimming process.

Software: FastQC 0.11.2 (2).

Cmd: fastqc File.fastq

- Input

File.fastq. One of output files of Trimmomatic.

- Output

A file in HTML format with statistical descriptive of reads quality and its associated compressed directory.

#### 1 .2. Contigs assembly and its quality

1.2.1. Contigs assembly

Software: Trinity V2.0.6 (3).

Cmd: Trinity --seqType fq --max_memory 50G --left Reads1.fastq --right

Reads2.fastq --CPU 6 --SS_lib_type RF

- Input
- Reads1.fastq, Reads2.fastq paired-en reads.
- Output
- Trinity.fasta. Contigs.
- Parameters
- seqType fq. Reads in format fastq
- CPU 6. Trinity can use 6 CPUs or threads.
- SS_lib_type RF. Option used only for paired-end strand-specific. This indicates that the first file is forward and the second is reverse

1.2.2. Evaluation of the quality of the assembly.

To evaluate the quality of contigs assembly, the amount of paired-end reads correctly aligned to the contigs was evaluated with Subread (4) and Bowtie (5).

Software: Subread 1.4.6 (4).

The software Subread makes the mapping in two steps, in the first step it builds an index for the sequences obtained with Trinity.

Cmd: subread-buildindex -o my_index Contigs.fasta

- Input
- Contigs.fasta. Contigs assembled by Trinity in fasta format.
- Output
- my_index. index of contigs.

In the second step Subread maps the reads to the contigs.

Cmd: subread-align -B 3 -T 5 -i my_index -r Read1.fastq -R Read2.fastq -o Output.sam

- Input
- Read1.fastq, Read2.fastq. Paired-end reads.
- Output
- Output.sam. Alignments in SAM format.
- Parameters
- B 3. Reports the top three mappings for reads
- T 5. Use 5 threads or CPUs.
- i my_index. Index of the contigs.

Software: Bowtie 0.12.9 (5).

The alignment of reads to contigs with Bowtie in Trinity also involves two steps. Alignment with script bowtie_PE_separate_then_join.pl.

Cmd: bowtie_PE_separate_then_join.pl --seqType fq --left Reads1.fastq --right Reads2.fastq --target Trinity.fasta --aligner bowtie -p 6 --all --best --strata -m 300

- Input
- Read1.fastq, Read2.fastq. Paired-end reads.
- Output
- Creates a directory with the mapping of the reads to the contigs in bam format.
- Parameters
- --aligner bowtie. mapping with Bowtie software.
- p 6. Use 6 threads.
- --all --best --strata**.** Report the best alignment for the pair of reads.
- m 300. The results for a pair of reads that have more than 300 reportable alignments are not reported.

Cmd: SAM_nameSorted_to_uniq_count_stats.pl ./bowtie_out/bowtie_out.nameSorted.bam > Mapping.Statistics.txt

- Input
- bowtie_out.nameSorted.bam. Mapping of reads to contigs in BAM format.

- Output

- Mapping.Statistics.txt. In this file is indicated the percentage of paired-end properly aligned

#### New Contigs.

Software: BRANCH (6).

Cmd: BRANCH --read1 Reads1.fastq --read2 Reads2fastq --transfrag Contigs.fasta --contig mRNA.Reference.Species.fasta --transcript Output.fasta

- Input
- Reads1.fastq, Reads2fastq. Paired-end reads.
- Contigs.fasta. Contigs assemble by Trinity.
- mRNA.Reference.Species.fasta. mRNA of reference species.
- Output
- Output.fasta. Contigs assembled by BRANCH.
- Parameters

BRANCH was used with default parameters.

## Step 2.

#### 2.1. Removal of redundant sequences.

Software: cd-hit-est V4.6.4 (7).

Cmd: cd-hit-est -i Contigs.fasta -o Output -c 1.0 -n 10 -d 0 -M 16000

- Input
- Contigs.fasta. The contigs assembly by Trinity and BRANCH.
- Output
- Output.clstr. Clusters of identical sequences.
- Parameters
- c 1.0. 100% of identity.
- n 10. word size 10.
- d 0. The sequence identifier stops in the first space.
- M 16000. 16000 MB limit of memory.

#### 2.2. Translation of contigs to polypeptide sequences.

Software: FrameDP V1.2.2 (8).

Cmd: /FrameDP.pl --cfg File.cfg --infile Contigs.fasta --outdir /Path/Directory.Output/

- Input.
- Contigs.fasta. Contigs after remotion of redundant sequences with cd-hit-est.
- Output.
- FrameDP creates multiple output files in one directory for each of the contigs. We retrieve afile in GFF3 format that this software produces for protein sequences.

FrameDP uses the configuration file File.cfg. There are several parameters that can be manipulated in this file as explained in (8), for the current analysis we worked with the default parameters and using as reference database the UniProtKB viridiplantae proteins (9) and the ncbi annotation of *Elaeis guineensis* (10), *Phoenix dactylifera* (11) and *Musa acuminata* (12).

Be Careful, FrameDP can create a lot of empty files, this command can to remove them.

Cmd: find <directory> -empty -type f -delete

### Step 3.

#### 3.1 Protein alignment.

Software: BLASTP (13).

- Input
- Sequences of proteins obtained by FrameDP in fasta format.
- Output
- Alignment in XML format.
- Parameters

- outfmt 5 (output in XML format).

### Step 4 and 5.

Steps 4 and 5 of the methodology are done by the main.pl perl script. This script processes the BlastP alignments, identifies chimeric and redundant polypeptides, and performs the scaffolding of the fragmented polypeptides. The following line is an example of how to use this script in linux:

Cmd: ./main.pl -b BlastP.alignment.xml -f polypeptides.faa -i 0.80 -q 0.60 -r 0.70 -s Output

- Input
- polypeptides.faa. Polypeptides sequences in fasta format.
- Output

There are 4 output files:

- Output.alignment.parameters: Identity and coverage between the polypeptides of the target / query species and the proteins of the reference / subject species.
- Output.chimeric_polypeptides: List of chimeric sequences.
- Output.table: The first column contains the identifiers of the species reference proteins, the second contains the polypeptides of the target species on which the scaffolding was performed if there are more of one sequence and the third column contains the identifiers of the redundant sequences.
- Output.fasta. It is a fasta file with the polypeptide products (PP) of the methodology.
- Parameters.
- b. BlastP alignment in format XML.
- f. Polypeptides of target species in fasta format.
- i. Minimum identity required in the alignment between the target species polypeptide and the reference species proteins.
- q. Minimum coverage required of the target species polypeptide when the coverage of reference species proteins is greater than 0.40.
- r. Minimum coverage required of the target species polypeptide when the coverage of reference species proteins is less than 0.40.
- s. Prefix of output files.

All the scripts used by main.pl and a readme that describes in detail the use and the required perl packages could be recovered from:

http://palm-comparomics.southgreen.fr/Download.html/Scaff.RRed.tar.gz

## Data such as Contigs, Polypeptides and PPs.

###

We give a brief description of the files with the main results of our work that can be retrieved from the website: [http://palm-comparomics.southgreen.fr/Download.html](http://palm-comparomics.southgreen.fr/Download.html/).

### Step 1.

- dwarf.embryo.denovo.fna. 264,349 contigs assembled by Trinity from SRX534380 reads (14).
- dwarf.embryo.new.fna. 20,847 contigs assembled by BRANCH from SRX534380 reads and *E. guineensis* mRNA.
- dwarf.endosperm.denovo.fna. 185,242 contigs assembled by Trinity from SRX518095 reads (14).
- dwarf.endosperm.new.fna. 29,122 contigs assembled by BRANCH from SRX518095 reads and *E. guineensis* mRNA.
- dwarf.leaf.denovo.fna. 88,707 contigs assembled by Trinity from SRX534428 reads (14).
- dwarf.leaf.new.fna. 27,625 contigs assembled by BRANCH from SRX534428 reads and *E. guineensis* mRNA.
- tall.denovo.fna. 89,133 contigs assembled by Trinity from SRX198908 reads (15).
- tall.new.fna. 23,283 contigs assembled by BRANCH from SRX198908 reads and *E. guineensis* mRNA.

### Step 2.

- Contigs.fna. 591,511 contigs represent the clusters found with cd-hit-est in the sequences obtained with Trinity and BRANCH.
- Polypeptides.Contigs.faa. 439,300 polypeptides translated by FrameDP from the nucleotide contigs.

### Step 5.

- Coconut.Proteins.faa. 29,366 coconut proteins product of workflow.

# References

1. Bolger AM, Lohse M, Usadel B. Trimmomatic: a flexible trimmer for Illumina sequence data. Bioinformatics. 2014 Jan;30(15):2114–20.
2. Andrews S. (2010). FastQC: a quality control tool for high throughput sequence data. Available from:<http://www.bioinformatics.babraham.ac.uk/projects/fastqc>.
3. Grabherr MG, Haas BJ, Yassour M, Levin JZ, Thompson DA, Amit I, et al. Full-length transcriptome assembly from RNA-Seq data without a reference genome. Nat Biotechnol Nature Biotechnology. 2011;29(7):644–52.
4. Liao Y, Smyth GK, Shi W. The Subread aligner: fast, accurate and scalable read mapping by seed-and-vote. Nucleic Acids Research. 2013 Apr;41(10).
5. Langmead B, Trapnell C, Pop M, Salzberg SL. Ultrafast and memory-efficient alignment of short DNA sequences to the human genome. Genome Biology. 2009;10(3):R25.
6. Bao E, Jiang T, Girke T. BRANCH: boosting RNA-Seq assemblies with partial or related genomic sequences. Bioinformatics. 2013;29(10):1250–9.
7. Li W, Jaroszewski L, Godzik A. Clustering of highly homologous sequences to reduce the size of large protein databases. Bioinformatics. 2001;17(3):282–3.
8. Gouzy J, Carrere S, Schiex T. FrameDP: sensitive peptide detection on noisy matured sequences. Bioinformatics. 2009;25(5):670–1.
9. Consortium TU. UniProt: a hub for protein information. Nucleic Acids Research. 2015;43(D1):D204–12.
10. National Center for Biotechnology Information [Internet]. National Center for Biotechnology Information. U.S. National Library of Medicine; [cited 2016Jun16]. Available from: ftp://ftp.ncbi.nih.gov/genomes/Elaeis_guineensis/
11. National Center for Biotechnology Information [Internet]. National Center for Biotechnology Information. U.S. National Library of Medicine; [cited 2016Jun16]. Available from:

ftp://ftp.ncbi.nih.gov/genomes/Phoenix_dactylifera/

1. National Center for Biotechnology Information [Internet]. National Center for Biotechnology Information. U.S. National Library of Medicine; [cited 2016Jun16]. Available from: ftp://[ftp.ncbi.nih.gov/genomes/Musa_acuminata/](http://ftp.ncbi.nih.gov/genomes/Musa_acuminata/).
2. Altschul SF, Gish W, Miller W, Myers EW, Lipman DJ. Basic local alignment search tool. Journal of Molecular Biology. 1990 Oct 5;215(3):403–10.
3. Huang Y-Y, Lee C-P, Fu JL, Chang BC-H, Matzke AJM, Matzke M. De Novo Transcriptome Sequence Assembly from Coconut Leaves and Seeds with a Focus on Factors Involved in RNA-Directed DNA Methylation. G3: Genes|Genomes|Genetics. 2014 Nov;4(11):2147–57.
4. Fan H, Xiao Y, Yang Y, Xia W, Mason AS, Xia Z, et al. RNA-Seq Analysis of *Cocos nucifera*: Transcriptome Sequencing and De Novo Assembly for Subsequent Functional Genomics Approaches. PLoS ONE. 2013 Mar 29;8(3):e59997.
